# Metabolically Healthy Overweight and Obesity, Transition to Metabolically Unhealthy Status and Cognitive Function: Results from the Framingham Offspring Study

**DOI:** 10.3390/nu15051289

**Published:** 2023-03-05

**Authors:** Matina Kouvari, Nathan M. D’Cunha, Thomas Tsiampalis, Manja Zec, Domenico Sergi, Nikolaj Travica, Wolfgang Marx, Andrew J. McKune, Demosthenes B. Panagiotakos, Nenad Naumovski

**Affiliations:** 1 Discipline of Nutrition and Dietetics, Faculty of Health, University of Canberra, Canberra, ACT 2601, Australia; 2 Functional Foods and Nutrition Research (FFNR) Laboratory, University of Canberra, Bruce, Ngunnawal Country, ACT 2617, Australia; 3 Department of Nutrition and Dietetics, School of Health Science and Education, Harokopio University, 17671 Kallithea, Attica, Greece; 4 School of Nutritional Sciences and Wellness, University of Arizona, Tucson, AZ 85721, USA; 5 Department of Translational Medicine, University of Ferrara, 44121 Ferrara, Italy; 6 Food & Mood Centre, The Institute for Mental and Physical Health and Clinical Translation, School of Medicine (IMPACT), Deakin University, Barwon Health, Geelong, VIC 3220, Australia; 7 Research Institute for Sport and Exercise, University of Canberra, Canberra, ACT 2601, Australia; 8 Discipline of Biokinetics, Exercise, and Leisure Sciences, School of Health Sciences, University of KwaZulu Natal, Durban 4000, South Africa

**Keywords:** metabolically healthy obesity, cognition, metabolic syndrome, obesity, healthy aging, metabolism

## Abstract

Aims: To evaluate the association between metabolically healthy overweight/obesity (MHO) status and longitudinal cognitive function while also considering the stability of the condition. Methods: In total, 2892 participants (mean age 60.7 (9.4) years) from Framingham Offspring Study completed health assessments every four years since 1971. Neuropsychological testing was repeated every four years starting from 1999 (Exam 7) to 2014 (Exam 9) (mean follow-up: 12.9 (3.5) years). Standardized neuropsychological tests were constructed into three factor scores (general cognitive performance, memory, processing speed/executive function). Healthy metabolic status was defined as the absence of all NCEP ATP III (2005) criteria (excluding waist circumference). MHO participants who scored positively for one or more of NCEP ATPIII parameters in the follow-up period were defined as unresilient MHO. Results: No significant difference on the change in cognitive function over time was observed between MHO and metabolically healthy normal weight (MHN) individuals (all *p* > 0.05). However, a lower processing speed/executive functioning scale score was observed in unresilient MHO participants compared to resilient MHO participants (β = −0.76; 95% CI = −1.44, −0.08; *p* = 0.030). Conclusions: Retaining a healthy metabolic status over time represents a more important discriminant in shaping cognitive function compared to body weight alone.

## 1. Introduction

Global obesity prevalence ranges from 11% to 15% and the results of the Non-Communicable Disease Collaboration analyses indicate the prevalence of obesity in the world doubled between 1975 and 2016 [1]. Excess fat accumulation, especially visceral adiposity, is linked to several chronic diseases, disability and reduced life expectancy and quality of life with a large body of research showing that obesity increases the risk of developing individual cardiometabolic diseases, as well as cardiometabolic multi-morbidity [2]. Recent estimates forecast a threefold increase in the number of dementia cases globally by 2050, underscoring the need for public health planning efforts and policy to address the needs of groups at higher risk for dementia [3].

Metabolically healthy overweight/obesity (MHO) affects approximately 10–15% of overweight or obese individuals. Recent findings from a vascular health perspective corroborate that metabolically benign obesity may not be an innocuous low-risk condition as previously believed [4,5,6]. In particular, two major issues have recently arisen. First, the definition of MHO status is still debated. The majority of prospective studies define MHO as a condition that does not meet the diagnostic criteria of metabolic syndrome. Hence, in many cases, individuals with obesity and even two metabolic abnormalities could be misclassified as being “healthy” [4,5,6]. In an effort to standardize the definition of MHO, a study by *Lavie* and colleagues [7] proposed a harmonized definition of MHO, moving from the more flexible concept of “metabolic syndrome absence” to a more comprehensive rationale that demands the absence of all metabolic syndrome features excluding waist circumference. Second, the stability of MHO status over time (i.e., no transition from metabolically healthy to metabolically unhealthy status) and its significance in defining future health outcomes remains to be fully elucidated. These points have recently been the subject of intense debate in the CVD research [4,5,6,7,8,9,10].

Evidence on the relationship between increased body weight, cognitive function, and dementia from prospective studies remain controversial, reporting neutral, adverse and age-dependent associations [11,12]. Several studies have observed an association between MHO and impaired cognitive function, as well as dementia [13,14,15]. However, to the best of our knowledge, no previous study has investigated the relationship between cognitive function, the aforementioned strictest definition of MHO [7] and the stability of this condition longitudinally. Using data from the Framingham Offspring Study [16], we aimed to determine how a priori defined MHO status using the latest criteria [7] is associated with cognitive function in this well-characterized cohort of community-dwelling adults. We posed two a priori research hypotheses: a. MHO status is not associated with poorer cognitive function over time compared to MHN status; b. The transition from MHO to metabolically unhealthy status (non-persistent MHO participants) is associated with poorer cognitive function compared to their resilient MHO counterparts.

## 2. Materials and Methods

### 2.1. Sample

The Framingham Heart Study (FHS) is a community-based prospective cohort study established in 1948 with the aim to identify risk factors that contribute to cardiovascular disease. More details on FHS can be found elsewhere [17]. In the current study, participants were members of the Offspring Cohort, which includes biological children of the original FHS cohort and spouses of offspring (*n* = 5124) who have undergone over nine health examination cycles approximately every 4 years since 1971. The present sample is based on the 2893 offspring participants who also underwent neuropsychological assessments starting on the seventh assessment in 1999. Follow-up data up to 2014 (Exam 9) were included in these analyses. Mean follow-up time was 12.9 (3.5) years. The protocol was approved by the Institutional Review Board of Boston University Medical Center, and all participants provided written informed consent. Access to the database was also approved by the University of Canberra Human Research Ethics Committee (UCHREC-2021–9271).

### 2.2. Neuropsychological Assessment

A standardized neuropsychological battery of tests was administered in three separate waves of testing. We constructed factor scores using the data from the battery to represent general cognitive performance, processing speed/executive function and memory in the same way as previously described by Bangen et al. (2019) [17]. In summary, factor scores were estimated from a 2-parameter logistic graded response item theory model of the neuropsychological test battery for each domain. The neuropsychological measures used to construct the memory, processing speed/executive function and general cognition factors are previously described [17]. In particular, for the memory factor, the Wechsler Memory Scale (WMS) Logical Memory immediate recall, delayed recall and recognition were used [18]. Trail Making Tests A and B, WMS Digit Span Backward, Wechsler Adult Intelligence Scale (WAIS) Similarities subtest, Controlled Word Association Test (FAS) and Category Fluency (Animals) were used to construct processing speed/executive function factor [19]. All the above variables combined with WMS Paired Associates total learning, learning of easy pairs, learning of hard pairs and delayed recall of hard pairs were used for the general cognition factor [20]. Each composite factor was scaled to have a mean of 50 and standard deviation (SD) of 10.

### 2.3. Combined Weight and Metabolic Status Definition

Normal weight was defined as body mass index (BMI) between 18.5 and 25 kg/m^2^, overweight as BMI from 25 to 29.9 kg/m^2^ and obesity as BMI ≥ 30 kg/m^2^. Underweight was defined as BMI < 18.5 kg/m^2^. Metabolic status was defined using the criteria suggested by Lavie and colleagues [7]. In particular, healthy metabolic status was defined as the absence of all of the following metabolic syndrome features such as hypertension, dyslipidemia and glycemic abnormalities. Hypertension was defined as systolic blood pressure (SBP) ≥ 130 mmHg and/or diastolic blood pressure (DBP) ≥ 85 mmHg [7]. Dyslipidemia was defined as triglyceride levels ≥ 150 mg/dL and/or high-density lipoprotein cholesterol (HDL-cholesterol) levels < 40 mg/dL in men and <50 mg/dL in women [7]. Glycemic abnormalities were defined as fasting glucose ≥ 100 mg/dL [7]. Medication treatments for the aforementioned conditions were set as alternative indicators of metabolic abnormalities. For the scope of the present work, participants were divided into four groups as follows; a. MHN defined as BMI < 25 kg/m^2^ and healthy metabolic status; b. MHO defined as BMI ≥ 25 kg/m^2^ and healthy metabolic status; c. MUN defined as BMI < 25 kg/m^2^ and unhealthy metabolic status; d. MUO defined as BMI ≥ 25 kg/m^2^ and unhealthy metabolic status.

### 2.4. Statistical Analysis

Baseline participant characteristics are presented in terms of mean (SD) values for continuous variables, and absolute (N) and relative (%) frequencies for categorical variables. Comparison among the different categories of obesity and metabolic health status was based on the one-way Analysis of Variance (ANOVA) for continuous characteristics and on the Pearson Chi-square test for categorical characteristics. An independent samples Student’s *t*-test was used to examine the difference in participant baseline characteristics (continuous) between those who remained resilient at the metabolically healthy obese status and those who transitioned to the metabolically unhealthy obese status. In addition, mixed effects linear regression analysis (both unadjusted and adjusted for participants characteristics) was implemented to examine the effect of the baseline obesity and metabolic health status on cognitive time trajectories. The same statistical methodology was also used to investigate the association of the transition to metabolically unhealthy status with cognitive time trajectories. All statistical analyses were performed using STATA software (version 14.0) and the statistical significance was set at a *p*-value < 0.05.

## 3. Results

### 3.1. Participants Characteristics Based on Their Obesity and Metabolic Health Status at Baseline

Table 1 presents participant demographic and baseline characteristics, both for the total sample, as well as separately according to their obesity and metabolic health status. Only participants with available responses on both metabolic and psychological assessment were included—excluding participants who were classified as MHN at the recruitment phase, yet they transitioned to other BMI or metabolic categories within the decade, as well as participants initially classified as MHO who changed BMI category within the follow-up period—for a final sample size of *n* = 1990. The sample was on average 60.7 (9.4) years old, and over half were women (54.2%). In total, 13.6% of the sample were metabolically healthy normal-weight, 16.7% were metabolically unhealthy normal-weight, 11.8% were metabolically healthy overweight/obese while the majority (57.9%) of the sample were metabolically unhealthy overweight/obese. Regarding cognitive factor scores, MHN participants seemed to have the best scoring in all metrics followed by their MHO counterparts while the unhealthy categories scored significantly lower (all *p*-values < 0.05).

Table 2 presents participant demographic and baseline characteristics for the MHO participants, both overall and separately for those who retained or lost their healthy metabolic status over the observation period. No significant differences between the two categories were observed other than the metabolic syndrome components (all *p*-values < 0.001).

Based on the results from the multivariable mixed effects linear regression analysis (Table 3), after adjusting for participants’ sex, baseline age, waist circumference, LDL-C levels, educational level and smoking status, when compared to MHN participants, a significant decrease in general cognitive performance scale score was observed among the MUN (β = −1.23; 95% CI = −2.00, −0.46; *p* = 0.002). There was no significant difference between MHN and MHO participants (*p* = 0.725). In the MUO participants, from Model 1 to Model 3, there was a significantly lower general cognitive performance scale score compared with MHO participants; however, this was not significant after adjusting for lipidemic and visceral adiposity factors (*p* = 0.283). Regarding the change observed in the memory scale score, when compared to the MHN reference group, lower scores were observed, both among the MUO (β = −1.48; 95% CI = −2.76, −0.19; *p* = 0.025), as well as among the MUN subjects (β = −2.89; 95% CI = −4.14, −1.64; *p* < 0.001), while there was no significant difference with the MHO participants (*p* = 0.813). Finally, regarding the change in the processing speed/executive functioning scale score, no significant difference was observed among all subsamples (all *p*-values > 0.05).

Based on the results from the multivariable mixed effects linear regression analysis (Table 4), after adjusting for participants’ sex, baseline age, waist circumference, LDL-C levels, educational level and smoking status, when compared to the participants who remained resilient in the MHO, a lower processing speed/executive functioning scale score was observed among those who transitioned to a metabolically unhealthy status (non-resilient MHO participants) (β = −0.76; 95% CI = −1.44, −0.08; *p* = 0.030). However, regarding the change observed in the memory score (*p* = 0.181), as well as in the general cognitive performance scale score (*p* = 0.722), there was no significant difference between the two categories.

The present work revealed that MHO status may not result in poorer cognitive function over time compared with MHN individuals. However, MHO status is a transient condition. Based on the findings of this study, overweight and obesity status resulted in poorer general cognitive performance. MHO participants who lost their metabolically healthy status seemed to have lower processing speed/executive functioning scores compared with their resilient MHO counterparts while no significant trends were observed in the general cognitive performance and memory scale scores. To the best of our knowledge, this is one of the first studies that examined the role of MHO status on cognitive trajectories using a stricter definition for metabolic status and considering the stability of this condition.

The paradoxical association between weight status and cognitive function is highly discussed in the literature. Previous studies suggest that being overweight or obese in older age is protective against the development of cognitive impairment or dementia [21,22,23,24,25]. In contrast, other studies have revealed that abnormal weight status in midlife is associated with poorer cognitive function over time resulting in twice as high risk of dementia compared with their normal weight counterparts [26,27]. In line with the aforementioned contradictions, a recent meta-analysis of longitudinal studies suggests a positive association between obesity in midlife and later dementia. Nevertheless, the opposite happens in case of obesity in older age, suggesting a potential protective effect of the maintenance of body weight [12].

The vast majority of previous works that examined the effect of weight status on cognitive function [28] did not stratify the sample according to their metabolic profile. Considering that unhealthy metabolic status and its specific features have been independently associated with cognitive disorders, the effect of overweight and obesity should be examined independently from and in the absence of metabolic abnormalities [28]. Here, we show that being overweight or obese *per se* may not be linked with cognitive decline especially in the context of no metabolic abnormalities. Several studies have reported that the presence of metabolic syndrome is a risk factor for mild cognitive impairment [29], AD [30] and vascular dementia [31]. In addition, another analysis of the Framingham Offspring Cohort revealed that metabolic syndrome (yet not defined with the strict definition used here) was associated with a lower level of cognitive function, implying higher rates of dementia [17]. However, all aforementioned studies did not stratify the sample according to their weight status. Nevertheless, a combined analysis of cohorts from Europe, the US and Asian countries (*n* = 1,349,857) found a harmful effect of higher BMI over twenty years before a dementia diagnosis, but lower BMI was predictive of dementia when BMI was assessed less than ten years before diagnosis [32]. Together, these findings imply that weight beyond the normal range may support cognitive function in older age.

As previously mentioned, a limited number of studies have examined the combined effect of weight and metabolic status on cognitive function, reporting controversial results with regard to the relationship between MHO and cognitive function. In line with the outcomes presented here, a longitudinal nationwide study using data from South Korea revealed that after a median follow-up time of 5.5 years, MHO individuals had the lowest incidence of overall dementia and AD compared to all other categories except for vascular dementia [33]. Similarly, results from the Worldwide Alzheimer’s Disease Neuroimaging Initiative revealed that MHO participants in older age had a lower risk for AD during the follow-up period [13]. In contrast, a cross-sectional analysis, again in a South Korean population revealed no association between MHO status and cognitive disorders [34].

The potential advantage of MHO status compared with MHN group has been ascribed to a variety of factors. Firstly, lower weight in older age is frequently associated with other comorbidities like cardiometabolic disorders, and an accelerated decline in BMI during older age often precedes cognitive impairment [35]. Secondly, adipokines secreted from the adipose tissue may also mediate this association [36]. In particular, a higher circulating leptin level results in a higher cerebral brain volume, which is inversely correlated with cognitive impairment [36]. Third, decreased serum IGF-1—observed in individuals with lower weight—was identified as an independent risk factor for AD and vascular dementia [37].

Recently, it has been suggested that MHO status may be transient in nature. Prospective population-based studies have revealed that a considerable proportion, ranging between 33 and 52%, of MHO middle-aged individuals lose this status over time [38,39,40]. This comes in line with the present work suggesting an even worse condition in case of older adults (i.e., about two to three MHO individuals) lost their metabolically healthy status after a 12-year observation period. Such evidence implies that there are resilient and non-resilient individuals with MHO who may have differences in their health status over time. This generates the hypothesis that metabolic abnormalities may indicate a threshold of cumulative obesity exposure translated to health risk. Non-resilient MHO status has been evaluated in relation to cardiometabolic health, revealing either positive [9,39] or neutral [38] associations with CVD onset. Hence, another novelty is that this is the first study examining the connection between the stability of MHO status and cognitive function. Our analysis suggested that MHO individuals who lose their metabolically healthy status present worse cognitive function over time compared with their resilient MHO counterparts, yet not in all cognitive domains.

### 3.2. Strengths and Limitations

The main strength of the present work is that this is the first study that evaluated the transition of MHO to MUO status and their longitudinal associations with cognitive function. Additionally, we examined these associations using a strict definition regarding metabolic status. Other strengths include a large, well characterized, community-based sample with a prospective study design and an ongoing follow-up for over four decades. The implementation of a comprehensive neuropsychological assessment at multiple time points is another strength that increases the validity of the examined outcome. This study also has limitations. First, the principal hypothesis examined here was related with an intermediate condition. Most of intermediate forms of the disease do not strictly correspond to a well-defined phenotype. To this issue, even if the bias attributed to the transition to other BMI or metabolic status categories was partially avoided, misclassification of transitions cannot be precluded due to the extended interim periods between follow-up assessments. Second, data on other factors co-existing with abnormal weight status and simultaneously affecting cognition such as sleep quality, medication and so on were not available for the current analysis; however, we believe that considering these conditions or factors would make the effect of the non-resilient MHO status even stronger. Lastly, the study sample included predominantly white subjects, who were generally healthy and well-educated, which may affect generalizability.

## 4. Conclusions

An increased weight status in an older population has been suggested as a protective factor against cognitive decline. However, the fact that MHO individuals did not have a clear benefit compared with their normal weight counterparts along with the proven instability of this condition implies the need for modifications with the aim to retain a healthy metabolic status during aging. At the same time, body weight alone does not seem to be a key driver of cognitive impairment, which instead is driven by an impairment in metabolic health. Considering the multi-comorbidity in older age being common, as well as the intercorrelation between cardiometabolic and brain health, retention of healthy metabolic status should be prioritized irrespective of weight status.

## Figures and Tables

**Table 1:** Baseline characteristics of the study sample according to the obesity and metabolic health status.

	Total Sample (N = 1990)	MHN (N = 271)	MUN (N = 333)	MHO (N = 234)	MUO (N = 1152)	*p*-Value
Demographic characteristics						
Age at baseline examination [in years; Mean (SD)]	60.7 (9.4)	57.3 (9.0)	63.4 (9.8)	57.3 (8.1)	61.4 (9.2)	<0.001
Women, *n* (%)	1079 (54.2)	213 (78.6)	204 (61.3)	125 (53.4)	537 (49.6)	<0.001
Education group, *n* (%)						
<High school	76 (3.8)	3 (1.1)	17 (5.1)	6 (2.6)	50 (4.3)	<0.001
High school	613 (30.8)	77 (28.4)	97 (29.1)	47 (20.2)	392 (34.0)	
Some college	495 (24.9)	59 (21.8)	90 (27.0)	72 (30.9)	274 (23.8)	
≥Some college	805 (40.5)	132 (48.7)	129 (38.7)	108 (46.4)	436 (37.8)	
Smoker at baseline examination, *n* (%)	215 (10.8)	37 (13.7)	45 (13.5)	28 (12.0)	105 (9.1)	0.036
Metabolic Syndrome components, *n* (%)						
Ever had glycaemic abnormality	1158 (67.1)	79 (33.1)	185 (66.8)	93 (45.6)	801 (79.5)	<0.001
Ever had hypertension	1152 (64.3)	101 (40.4)	202 (68.9)	112 (51.9)	737 (71.4)	<0.001
Ever had dyslipidaemia	767 (45.9)	31 (13.1)	100 (39.2)	45 (22.3)	591 (60.4)	<0.001
Cognitive function at baseline, Mean (SD)						
General Cognitive Performance	43.3 (4.8)	44.3 (5.1)	42.7 (5.2)	43.7 (4.3)	43.1 (4.7)	<0.001
Memory	49.5 (7.9)	51.7 (7.3)	48.5 (8.7)	50.8 (7.6)	49.0 (7.7)	<0.001
Processing Speed/Executive Functioning	40.4 (4.1)	40.1 (4.1)	40.5 (4.2)	39.8 (2.9)	40.5 (4.3)	0.055

**Notes**: MHN = Metabolically healthy normal-weight; MUN = Metabolically unhealthy normal-weight; MHO = Metabolically healthy overweight/obese; MUO = Metabolically unhealthy overweight/obese; *p*-value was given by the one-way ANOVA in case of the continuous characteristics and by the Pearson Chi-square test in case of the categorical characteristics; Cognitive scores reflect performance at first cognitive assessment and are internally scaled within the Framingham Offspring Study; Healthy metabolic status was defined as the absence of hypertension, dyslipidemia and glycaemic abnormalities; Hypertension was defined as systolic blood pressure ≥ 130 mm Hg and/or diastolic blood pressure ≥ 85 mm Hg or use of anti-hypertensive medication. Dyslipidemia was defined as triglyceride levels ≥ 150 mg/dL and/or high-density lipoprotein levels < 40 mg/dL in men and <50 mg/dL in women or use of statins. Glycaemic abnormalities were defined as fasting glucose ≥ 100 mg/dL or use of anti-diabetics; MHN defined as BMI < 25 kg/m^2^ and healthy metabolic status; MHO defined as BMI ≥ 25 kg/m^2^ and healthy metabolic status; MUN defined as BMI < 25 kg/m^2^ and unhealthy metabolic status; MUO defined as BMI ≥ 25 kg/m^2^ and unhealthy metabolic status. Participants’ characteristics of the MHO participants and whether they remained resilient or transitioned to an unhealthy metabolically health status.

**Table 2:** Characteristics of the metabolically healthy overweight/obese participants (at baseline), both in total, as well as separately for those who remained resilient and for those who transitioned to an unhealthy metabolically health status.

	MHO (N = 230) *	Resilient (N = 66)	Non-Resilient (N = 164)	*p*-Value
Demographic characteristics				
Age at baseline examination [in years; Mean (SD)]	57.3 (8.1)	57.6 (6.9)	57.0 (8.5)	0.611
Women, *n* (%)	125 (53.4)	38 (57.6)	85 (51.8)	0.429
Education group, *n* (%)				
<High school	6 (2.6)	2 (3.0)	4 (2.5)	0.504
High school	47 (20.2)	13 (19.7)	33 (20.2)	
Some college	72 (30.9)	16 (24.2)	55 (33.7)	
≥Some college	108 (46.4)	35 (53.1)	71 (43.6)	
Smoker at baseline examination, *n* (%)	28 (12.0)	8 (12.1)	19 (11.6)	0.909
MetS components, *n* (%)				
Ever had glycaemic abnormality	93 (45.6)	0 (0.0)	92 (56.1)	<0.001
Ever had hypertension	112 (51.9)	0 (0.0)	112 (68.3)	<0.001
Ever had dyslipidaemia	45 (22.3)	0 (0.0)	45 (27.4)	<0.001
Cognitive function at baseline, Mean (SD)				
General Cognitive Performance	43.7 (4.3)	43.6 (5.5)	43.7 (3.7)	0.831
Memory	50.8 (7.6)	50.1 (7.4)	51.4 (7.3)	0.234
Processing Speed/Executive Functioning	39.8 (2.9)	40.2 (4.2)	39.4 (1.3)	0.144

**Notes**: * There was no available information for the change of obesity and metabolic health status for N = 4 participants; MHO = Metabolically healthy overweight/obese; *p*-value was given by the Independent samples *t*-test in case of the continuous characteristics and by the Pearson Chi-square test in case of the categorical characteristics; Cognitive scores reflect performance at first cognitive assessment and are internally scaled within the Framingham Offspring Study; Healthy metabolic status was defined as absence of hypertension, dyslipidemia and glycaemic abnormalities; Hypertension was defined as systolic blood pressure ≥ 130 mm Hg and/or diastolic blood pressure ≥ 85 mm Hg. Dyslipidemia was defined as triglyceride levels ≥ 150 mg/dL and/or high-density lipoprotein levels < 40 mg/dL in men and <50 mg/dL in women. Glycaemic abnormalities were defined as fasting glucose ≥ 100 mg/dL; MHN defined as BMI < 25 kg/m^2^ and healthy metabolic status; MHO defined as BMI ≥ 25 kg/m^2^ and healthy metabolic status.

**Table 3:** Results from the mixed- effects linear models regarding the association of baseline participants’ obesity and metabolic health status with cognitive time trajectories.

Reference Category: MHN	General Cognitive Performance	Memory Scale	Processing Speed/Executive Functioning Scale
Model 1: Crude model			
MUO	−1.08 (−1.71, −0.45) **	−2.72 (−3.74, −1.69) ***	−0.36 (−0.83, 0.10)
MHO	−0.67 (−1.50, 0.17)	−0.75 (−2.11, 0.60)	−0.41 (−1.03, 0.20)
MUN	−1.47 (−2.23, −0.70) ***	−3.34 (−4.58, −2.10) ***	−0.54 (−1.10, 0.02) *
Model 2: Adjusted for age, sex, educational level			
MUO	−0.76 (−1.40, −0.12) **	−2.05 (−3.09, −1.01) ***	−0.37 (−0.85, 0.10)
MHO	−0.42 (−1.25, 0.42)	−0.23 (−1.59, 1.13)	−0.42 (−1.04, 0.20)
MUN	−1.29 (−2.05, −0.53) **	−2.98 (−4.21, −1.74) ***	−0.54 (−1.11, 0.02) *
Model 3: Model 2 + Baseline smoking status			
MUO	−0.77 (−1.41, −0.13) **	−2.05 (−3.09, −1.00) ***	−0.38 (−0.86, 0.10)
MHO	−0.42 (−1.25, 0.42)	−0.23 (−1.59, 1.13)	−0.42 (−1.04, 0.20)
MUN	−1.29 (−2.05, −0.53) **	−2.98 (−4.22, −1.74) ***	−0.54 (−1.11, 0.02) *
Model 4: Model 3 + Baseline waist circumference + LDL-cholesterol levels			
MUO	−0.43 (−1.23, 0.36)	−1.48 (−2.76, −0.19) **	−0.29 (−0.77, 0.19)
MHO	−0.16 (−1.07, 0.75)	0.18 (−1.29, 1.65)	−0.36 (−0.98, 0.26)
MUN	−1.23 (−2.00, −0.46) **	−2.89 (−4.14, −1.64) ***	−0.51 (−1.08, 0.06) *

**Notes**: Results are presented in the form of beta- coefficients (95% Confidence Interval) and regard the difference of the metabolically healthy normal-weight participants with the rest categories of the participants’ obesity and metabolic health status; Healthy metabolic status was defined as absence of hypertension, dyslipidemia and glycaemic abnormalities; Hypertension was defined as systolic blood pressure ≥ 130 mm Hg and/or diastolic blood pressure ≥ 85 mm Hg. Dyslipidemia was defined as triglyceride levels ≥ 150 mg/dL and/or high-density lipoprotein levels < 40 mg/dL in men and <50 mg/dL in women. Glycaemic abnormalities were defined as fasting glucose ≥ 100 mg/dL; MHN defined as BMI < 25 kg/m^2^ and healthy metabolic status; MHO defined as BMI ≥ 25 kg/m^2^ and healthy metabolic status; MUN defined as BMI < 25 kg/m^2^ and unhealthy metabolic status; MUO defined as BMI ≥ 25 kg/m^2^ and unhealthy metabolic status *** *p* < 0.001; ** *p* < 0.05; * *p* < 0.01.

**Table 4:** Results from the mixed-effects linear models regarding the association of the transition to metabolically unhealthy status with cognitive time trajectories in overweight and obese participants (N = 230).

	General Cognitive Performance	Memory Scale	Processing Speed/Executive Functioning Scale
Model 1: Crude model			
Resilient vs. non-resilient	0.13 (−1.10, 1.36)	1.09 (−0.85, 3.03)	−0.78 (−1.45, −0.10) **
Model 2: Adjusted for age, sex, educational level			
Resilient vs. non-resilient	0.21 (−1.00, 1.43)	1.25 (−0.68, 3.18)	−0.76 (−1.44, −0.08) **
Model 3: Model 2 + Baseline smoking status			
Resilient vs. non-resilient	0.20 (−0.99, 1.40)	1.26 (−0.67, 3.19)	−0.76 (−1.45, −0.08) **
Model 4: Model 3 + Baseline waist circumference + LDL- cholesterol levels			
Resilient vs. non-resilient	0.22 (−1.01, 1.46)	1.31 (−0.62, 3.24)	−0.76 (−1.44, −0.08) **

**Notes:** Results are presented in the form of beta- coefficients (95% Confidence Interval) and regard the difference of the metabolically healthy normal-weight participants with the rest categories of the participants’ obesity and metabolic health status; Healthy metabolic status was defined as absence of hypertension, dyslipidemia and glycaemic abnormalities; Hypertension was defined as systolic blood pressure ≥ 130 mm Hg and/or diastolic blood pressure ≥ 85 mm Hg. Dyslipidemia was defined as triglyceride levels ≥ 150 mg/dL and/or high-density lipoprotein levels < 40 mg/dL in men and <50 mg/dL in women. Glycaemic abnormalities were defined as fasting glucose ≥ 100 mg/dL; MHN defined as BMI < 25 kg/m^2^ and healthy metabolic status; MHO defined as BMI ≥ 25 kg/m^2^ and healthy metabolic status; MUN defined as BMI < 25 kg/m^2^ and unhealthy metabolic status; MUO defined as BMI ≥ 25 kg/m^2^ and unhealthy metabolic status ** *p* < 0.05.

## Data Availability

Not applicable.
